# The peroxisome proliferator-activated receptor (PPAR) alpha agonist fenofibrate maintains bone mass, while the PPAR gamma agonist pioglitazone exaggerates bone loss, in ovariectomized rats

**DOI:** 10.1186/1472-6823-11-11

**Published:** 2011-05-26

**Authors:** Astrid K Stunes, Irene Westbroek, Björn I Gustafsson, Reidar Fossmark, Jan H Waarsing, Erik F Eriksen, Christiane Petzold, Janne E Reseland, Unni Syversen

**Affiliations:** 1 Department of Cancer Research and Molecular Medicine, Norwegian University of Science and Technology, NTNU, Trondheim, Norway; 2 Internal Medicine and Orthopaedics, Erasmus MC, Rotterdam, the Netherlands; 3 Department of Gastroenterology, St Olav's University Hospital HF, Trondheim, Norway; 4 Hormone Laboratory, Aker University Hospital, Oslo, Norway; 5 Department of Biomaterials, Institute for Clinical Dentistry, University of Oslo, Oslo, Norway; 6 Department of Endocrinology, St Olav's University Hospital HF, Trondheim, Norway

## Abstract

**Background:**

Activation of peroxisome proliferator-activated receptor (PPAR)gamma is associated with bone loss and increased fracture risk, while PPARalpha activation seems to have positive skeletal effects. To further explore these effects we have examined the effect of the PPARalpha agonists fenofibrate and Wyeth 14643, and the PPARgamma agonist pioglitazone, on bone mineral density (BMD), bone architecture and biomechanical strength in ovariectomized rats.

**Methods:**

Fifty-five female Sprague-Dawley rats were assigned to five groups. One group was sham-operated and given vehicle (methylcellulose), the other groups were ovariectomized and given vehicle, fenofibrate, Wyeth 14643 and pioglitazone, respectively, daily for four months. Whole body and femoral BMD were measured by dual X-ray absorptiometry (DXA), and biomechanical testing of femurs, and micro-computed tomography (microCT) of the femoral shaft and head, were performed.

**Results:**

Whole body and femoral BMD were significantly higher in sham controls and ovariectomized animals given fenofibrate, compared to ovariectomized controls. Ovariectomized rats given Wyeth 14643, maintained whole body BMD at sham levels, while rats on pioglitazone had lower whole body and femoral BMD, impaired bone quality and less mechanical strength compared to sham and ovariectomized controls. In contrast, cortical volume, trabecular bone volume and thickness, and endocortical volume were maintained at sham levels in rats given fenofibrate.

**Conclusions:**

The PPARalpha agonist fenofibrate, and to a lesser extent the PPARaplha agonist Wyeth 14643, maintained BMD and bone architecture at sham levels, while the PPARgamma agonist pioglitazone exaggerated bone loss and negatively affected bone architecture, in ovariectomized rats.

## Background

Peroxisome proliferator-activated receptors (PPARs) are ligand-activated nuclear receptors, and exist as three different subtypes in mammals (PPARα, PPARδ and PPARγ (with isoforms γ1, γ2 and γ3)) [1,2]. All PPARs form heterodimers with the 9-*cis*-retinoic acid receptor (RXR). PPARs are ubiquitously expressed, but with a tissue specific distribution, and are involved in the regulation in a broad specter of biological processes, particularly in carbohydrate and lipid homeostasis [2-5]. Endogenous ligands for PPARs include eicosanoids, fatty acids and fatty acid derivatives [3].

Because of their metabolic actions, PPARs (as yet preferentially PPARα and γ ) have become major drug targets [6]. Wyeth 14643 (pirinixic acid) is a potent PPARα receptor activator [7], but not in clinical use due to suspected liver toxicity [8]. Fenofibrate belongs to a class of fibrates which are mainly PPARα agonists, and is currently used for treatment of hypercholesterolemia and hypertriglyceridemia. Fibrates may also activate PPARγ and δ, but to a much lesser degree and this may also vary between the different fibrates [4,9]. Thiazolidinediones/glitazones are synthetic PPARγ agonists used for treatment of type 2 diabetes mellitus (T2DM) [2,3,10].

All PPARs are expressed in both human and rodent osteoblasts and osteoclasts, as reviewed by Giaginis *et al. *[11], and a role for the PPARs in the regulation of bone metabolism, has been proposed. Although the significance of PPARα and its agonists in bone metabolism remains poorly elucidated, we have demonstrated that administration of the PPARα agonist fenofibrate, increases femoral bone mineral density (BMD) and reduces medullary area in intact female rats [12,13], suggesting a positive impact on skeletal homeostasis. Recently, bezafibrate and linoleic acid which are PPARα/δ agonists [4], were shown to up regulate osteoblast differentiation and induce periosteal bone formation in intact male rats [9].

While the information on the role of PPARα and PPARδ in bone is sparse, more is known about the significance of PPARγ. *In vitro*, PPARγ agonists promote adipocyte differentiation preferentially over osteoblast differentiation [14-18]. PPARγ might act as a molecular switch driving differentiation of mesenchymal stem cells (MSC) into adipocytes, and PPARγ insufficiency enhances differentiation of osteoblast progenitors [19]. The role of PPARγ activation in osteoclasts is less characterized with several contradictory reports [20-23]. *In vivo *studies revealed elevated bone loss in rodents treated with the PPARγ agonists rosiglitazone [24-28] and darglitazone [29]. We have previously shown that the PPARγ agonist pioglitazone reduces whole body BMD and causes impairment of the mechanical strength in normal female rats [12,13]. Glitazones also induce bone loss in elderly women [30] and men [31] with T2DM. Furthermore, an increased fracture risk was observed in T2DM women treated with glitazones [32]. Even short-term glitazone treatment decreased bone formation and BMD in healthy, non-diabetic postmenopausal women [33].

In order to better characterize the role of PPARα in bone, we examined the long-term skeletal effects of the PPARα agonists fenofibrate and Wyeth 14643 in ovariectomized rats, and compared the effects to those of the PPARγ agonist pioglitazone. Bone effects were monitored using DXA and μCT. Additionally, plasma levels of osteocalcin and fragments of collagen type I, and the adipokines leptin and adiponectin, were analyzed.

## Methods

### Materials

Fenofibrate was a gift from Professor Rolf Berge at the University of Bergen, Norway, Wyeth 14643 was purchased from ChemSyn Laboratories, USA, and pioglitazone was kindly provided by Eli Lilly, Norway. Methylcellulose (M7140, Sigma-Aldrich, Norway) was used as vehicle in the *in vivo *study.

### Animals

The Animal Welfare Committee at St. Olav's University Hospital in Trondheim approved the study. Ovariectomy was used as a model-system for osteoporosis in rodents [34-36].

Fifty-five female Sprague Dawley rats, 12 weeks of age, (252 ± 16.4 g) were housed in wire-top cages with aspen woodchip bedding (B&K Universal Ltd, UK). Room temperature was 24 ± 1.0°C with a relative humidity of 40-50% and a twelve-hour light/dark cycle. Rat and Mouse Diet (B&K Universal Ltd) and tap water were provided *ad libitum*. The animals were randomly assigned to five groups of eleven rats. One group was sham-operated and given vehicle (SHAM). The other four groups were ovariectomized and given vehicle (OVX), fenofibrate (90.0 mg/kg) (FENO OVX), Wyeth 14643 (90.0 mg/kg) (WY OVX) or pioglitazone (35.0 mg/kg) (PIO OVX), respectively, by daily intragastric gavage. All agents were dissolved in methylcellulose. The doses of the different agents were chosen according to results obtained in other studies [37-39]. Administration of test substances was initiated one week after surgery, and lasted for four months. The rats were weighed at baseline and weekly throughout the study, and femoral and whole body DXA scans were performed in duplicates at baseline, and after two and four months.

Animals were anesthetized with 2.0 ml/kg body weight of a combination of fluanison (2.5 mg/ml), fentanyl (50 μg/ml), and midazolam (1.25 mg/ml), before surgery, DXA scans, and prior to sacrifice.

Blood samples were collected by cardiac puncture during final anaesthesia. After sacrifice, both femurs were dissected, weighed, lengths measured, and stored at -80°C or 4% formalin until analyzed. The livers were dissected and weighed.

### DXA measurements

Body weight (g), fat mass (g), lean mass (g), bone mineral content (BMC) (g), as well as whole body and femur BMD (g/cm^2^), were measured by DXA in anesthetized animals, using a Hologic QDR 4500A, and small animal software. The coefficient of variation (CV) was 2.4% for body weight, 2.2% for fat mass, 0.28% for lean mass, 0.54% for whole body BMC, 3.0% for femur BMC, 0.60% for whole body BMD and 0.71% for femur BMD. BMC and BMD are presented as % change from baseline.

### μCT measurements

The proximal femurs, including the femoral head and the metaphysis of the dissected formalin-fixed femurs were scanned in a SkyScan 1072 microtomograph (SkyScan, Antwerp, Belgium), with a voxelsize of 11.89 μm. Scans were processed, and three-dimensional morphometric analyses of the femurs were done using free software of the 3D-Calc Project (http://www.erasmusmc.nl/47460/386156/Downloads). Femoral head and metaphysis data sets were analyzed separately. Cortcial bone and trabecular bone were separated using in-house developed software. For each cross-section in the 3D dataset, a virtual mask of the total bone was created and used to identify the bone marrow regions in the original image. The marrow regions were expanded into a mask of the total marrow cavity using a close operation. Bone inside the total marrow cavity is considered trabecular bone, the remainder is regarded as cortex. Cortical bone volume (Ct.V, μm^3^), cortical thickness (Ct.Th, μm), trabecular bone volume (BV, μm^3^), total bone volume, the region of interest adjacent to the endocortical boundary including the trabecular bone as previously described [40] (TV, μm^3^), trabecular thickness (Tb.Th, μm), trabecular bone volume fraction (BV/TV), connectivity density (CD, 1/mm^3^) [41] and structure model index (SMI, (0-3)) [42] were determined. SMI indicates whether the trabeculae are more rod-like (SMI = 3) or more plate-like (SMI = 0), and values between 0 and 3 represent the volume ratio of rods and plates, analyzed as previously described [42].

### Biomechanical testing

The right femurs were thawed in Ringers^® ^solution before mechanical testing of the femoral neck and shaft was performed. The shafts were fractured 18.7 mm from the femoral condyles in three point cantilever bending as previously described [43]. The proximal femur was fixed in a clamp, the cam of the rotating wheel engaged the femoral condyles, and a fulcrum positioned 18.7 mm anteriorly from the condyles was the third point of force application. All tests were done at a loading rate of 0.095 radians/second (5.43°/second). The load in the test apparatus, a MTS 858 Mini Bionix^® ^Axial/Torsional Test System (MTS Systems Corporation, Minnesota, USA), was measured with a MTS Test Star TM Sensor Cartridge Force 250 N load cell and registered in MTS Test Star II software. Ultimate bending moment (M) was calculated as the ultimate load before failure multiplied by the moment arm by which the load was applied (Newton Meter, Nm). Energy absorption and stiffness were read directly or calculated from the computer recordings as previously described [43].

### Osteocalcin, CTx, leptin and adiponectin analyses in rat plasma

The amount of osteocalcin in plasma was determined by a Rat-MID osteocalcin enzyme-linked immunosorbent assay (ELISA) kit (Nordic Bioscience Diagnostics A/S, Denmark), according to the manufacturer's protocol. The detection limit was 50 ng/ml, and intra- and interassay variations were 5.0% and 5.5%, respectively. Bone resorption markers in plasma (CTx) were analyzed by a RatLaps ELISA kit (Nordic Bioscience Diagnostics A/S) according to the instructions from the manufacturer. The detection limit was 3.0 ng/ml, and intra- and interassay variations were 5.6% and 11%, respectively. Leptin and adiponectin were analyzed in rat plasma by radioimmunoassays (RIA) according to the manufacturer's protocol (Linco Research, USA). Detection limits were 3.0 pg/ml for leptin and 41 pg/ml for adiponectin. Intra- and interassay variations for the RIAs were < 14% and < 4.0%, respectively.

### Bone marrow cell preparation

Bone marrow cells were obtained from femurs and tibiae from three intact female Sprague Dawley rats (251 ± 4.5 g) by centrifugation as described by Dobson *et al. *[44]. Briefly, tibiae and femurs were removed and all soft tissue was removed. The proximal ends were cut off, and the bones briefly centrifuged (1000 g, 10 s). The bone marrow pellets from each animal were pooled and resuspended in culture medium and passed through a 21-gauge needle to achieve a single cell suspension.

### Mineralizing fibroblast-colony-forming unit cultures assays

For *in vitro *studies: Dulbecco's Modified Eagle Medium (DMEM) was supplemented with penicillin/streptomycin (10 U/ml), L-glutamine (0.1 mg/ml), sodium pyruvate (1.0 mM), (all from Gibco BRL Invitrogen, Paisley, UK). Mineralizing fibroblast-colony-forming unit (mCFU-f) cultures assays were performed essentially as previously described by Scutt *et al. *[45]. Nucleated bone marrow cells from three female rats were seeded in DMEM/10% fetal calf serum (FCS) (EuroClone, Devon, UK) with 10 nM dexamethasone (Sigma, Oslo, Norway), 50 μg/ml ascorbic acid (Sigma) and 2.0 mM β-glycerophosphate, in 6 well-plates (1.0 × 10^6 ^cells/well). Experiments were performed with cells from three female rats in three individual experiments, and with at least three parallel wells for each concentration of the drugs. Cells were treated with vehicle and different concentrations of the PPARα agonist fenofibrate and the PPARγ agonist pioglitazone (0.01-10 μM). The media were changed after 5 days, and thereafter every second day for 21 days. Cultures were terminated by washing with phosphate-buffered saline (PBS) and fixed by adding cold 100% ethanol. Wells were stained for alkaline phosphatase (ALP) by addition of 0.5 mg/mL napthol AS-BI phosphate (Sigma) and 1.0 mg/mL fast red B (Sigma) in Tris buffer (pH 7.5) for 30 minutes, washed with distilled water, photographed with a digital camera, and destained with 100% ethanol over night. Calcium-positive colonies were stained with 1.0 mg/mL alizarin red (Sigma) in distilled water adjusted to pH 5.5 with NH_3 _for 30 minutes, washed with distilled water, photographed, and destained with 5% perchloric acid for 5 minutes. Collagen-positive colonies were stained with 1.0 mg/mL sirius red (Sigma) in statured picric acid for 18 hours, washed with distilled water and photographed. Picture processing was performed in Adobe Photoshop software (Adobe, San Jose, CA, USA) and quantification of colonies was performed using the ImageQuant Software (Amersham Biosciences, Piscataway, NJ, USA).

### Statistical analyses

All measurements were performed in duplicates or triplets. Data are expressed as means ± SD or means ± SEM, as indicated in figures and tables. All data were tested for normality with the Shapiro-Wilk normality test. Normally distributed parameters were tested with two-tailed unpaired Student's t-test, or one-way ANOVA with Bonferroni's post test, while parameters that were not normally distributed were tested with Mann-Whitney's two tailed test, or Kruskal Wallis test with Dunn's post test. Significance was assumed at *p*-values lower than 0.05. Fat and lean mass are presented in % of body weight and whole body and femoral BMD and BMC data are presented as % change from baseline. Correlations between ultimate bending moment in the femoral neck and shaft and whole body and femoral BMD were analyzed with a two-way Spearman's Rank correlation test.

## Results

### General observations and body composition

**Figure 1:**
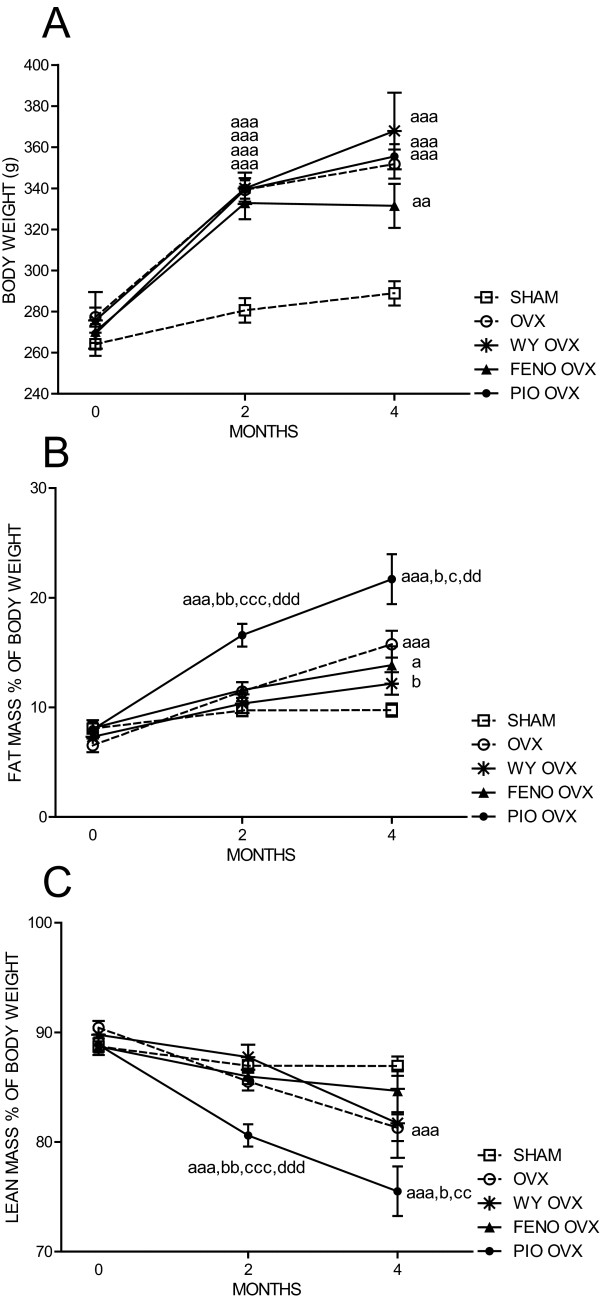
**Body weight (**A**), fat mass in % of body weight (**B**) and leans mass in % of body weight (**C**) at baseline, 2 and 4 months**. Rats were either sham operated and given vehicle (SHAM) or ovariectomized and given vehicle (OVX), Wyeth 14643 (WY OVX), fenofibrate (FENO OVX) or pioglitazone (PIO OVX) respectively. Data are presented as means ± SEM. ^a, aa,aaa ^= *p *< 0.05, *p *< 0.01, *p *< 0.001 compared to the SHAM, ^b,bb ^= *p *< 0.05, *p *< 0.01 compared to the OVX. ^c, cc,ccc ^= *p *< 0.05, *p *< 0.01, *p *< 0.001 compared to FENO OVX. ^dd,ddd ^= *p *< 0.01, *p *< 0.001 compared to WY OVX.

**Figure 2:**
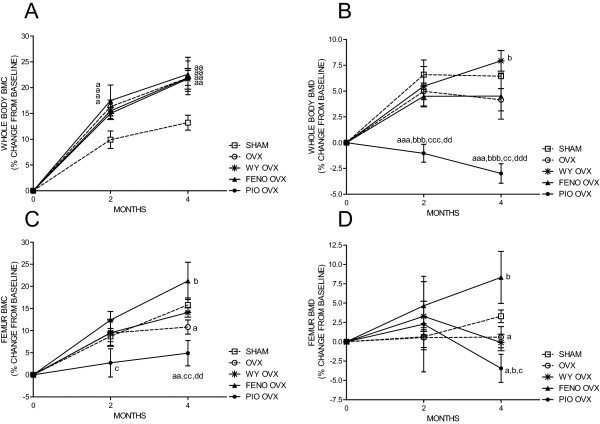
**Whole body BMC (**A**), whole body BMD (**B**), femoral BMC (**C**) and femoral BMD (**D**) in % change from baseline after 2 and 4 months**. Rats were either sham operated and given vehicle (SHAM) or ovariectomized and given vehicle (OVX), Wyeth 14643 (WY OVX), fenofibrate (FENO OVX) or pioglitazone (PIO OVX) respectively. Data are presented as means ± SEM. ^a,aa,aaa ^= *p *< 0.05, *p *< 0.01, *p *< 0.001 compared to SHAM. ^b,bbb ^= *p *< 0.05, *p *< 0.001 compared to OVX. ^c,cc,ccc ^= *p *< 0.05, *p *< 0.01, *p *< 0.001 compared to FENO OVX. ^dd,ddd ^= *p *< 0.01, *p *< 0.001 compared to WY OVX.

A total of seven rats died during the study, two rats in the WY OVX group, two in the PIO OVX group, and one rat in each of the other groups. There were no differences in body weight, lean or fat mass between the groups seven days after surgery, when administration of test substances was initiated. The body weight increased in all groups from baseline until the end of the study (Figure 1A). There was no difference in body weight between the four OVX groups during the study, but they all had significantly higher body weight than the SHAM group after two and four months (Figure 1A). All OVX groups except WY OVX had significantly higher % fat mass than the SHAM group after four months (Figure 2B), while the PIO OVX group had significantly higher % fat mass and lower % lean mass than all other groups after both two and four months (Figures 1B and 1C). The FENO OVX and the WY OVX had similar lean mass as SHAM, while the OVX controls had significantly lower lean mass than SHAM after four months (Figure 1C).

The length and weight of the femurs did not differ between the groups.

The liver weights (in % of total body weight) were higher in the WY OVX group (4.34 ± 0.61%) and the FENO OVX group (3.58 ± 0.58%), compared to the other groups; vs. SHAM; 2.34 ± 0.28% (*p *< 0.001 and *p *< 0.001), vs. OVX; 2.19 ± 0.09% (*p *< 0.001 and *p *< 0.001), and vs. PIO OVX; 2.18 ± 0.50%, (*p *< 0.001 and *p *< 0.001). We did not detect any liver pathology by gross visual inspection, however livers were not further examined, as this was not within the scope of this study.

In conclusion, all the ovariectomized groups had increased body weight compared to SHAM, while the PIO OVX had a significantly higher fat mass than all the other groups (Figure 1).

### Whole body and femoral BMC and BMD

There were no differences in whole body or femoral BMC or BMD between the groups seven days after surgery (at the beginning of administration). There was an increase in % change in whole body BMC from baseline in all groups throughout the study (Figure 2A). All ovariectomized groups had a significantly higher whole body BMC than the SHAM group after two and four months, but there was no significant difference in whole body BMC between the ovariectomized groups at any time (Figure 2A).

Whole body BMD increased in all groups, except for the PIO OVX group, where a decline from baseline was observed (Figure 2B). The WY OVX group had a significantly higher increase in whole body BMD than the OVX group, after four months.

After four months, an increase in femoral BMC was observed in all groups (Figure 2C). However, the change in femoral BMC for the OVX and PIO OVX groups were significantly lower than the SHAM group (Figure 2C), while maintained at SHAM levels in FENO OVX and WY OVX, and significantly higher in the FENO OVX group compared to OVX controls after four months (FENO OVX; +21.3 ± 4.2% vs. OVX; +10.0 ± 1.6%, *p *= 0.03) (Figure 2C).

The gain in femoral BMD from baseline was similar in the SHAM and FENO OVX groups, while no gain was observed in the OVX and WY OVX groups after four months. In the PIO OVX there was a decline in femoral BMD from baseline (Figure 2D). The increase in femoral BMD was significantly higher in the SHAM and the FENO OVX groups after four months compared to the OVX group (SHAM; +3.3 ± 0.8% vs. OVX; 0.0 ± 1.2%, *p *= 0.03 and FENO OVX; +8.3 ± 4.2% vs. OVX; 0.0 ± 1.2%, *p *= 0.03) (Figure 2D).

In summary, ovariectomized rats given fenofibrate and Wyeth maintained whole body and femur BMD at sham or ovariectomized controls levels, while the PIO OVX group had a pronounced decrease in BMD compared to all the other groups.

### Bone architecture

μCT analyses revealed significantly higher TV and SMI, and lower Ct.V, Ct.Th and BV/TV ratio in both the femoral heads and metaphyses in the OVX compared to the SHAM group (Tables 1 and 2). Furthermore, Tb.Th in the femoral head, and BV in the femoral metaphysis were significantly reduced in the OVX group compared to the SHAM group (Tables 1 and 2).

**Table 1:** Mean values ± SD of bone architecture parameters in the femoral head, determined by means of μCT scanning analyses in rats after 4 months of treatment

FEMORAL HEAD	SHAM (sham-operated controls) (N = 10)	OVX (ovariectomized controls) (N = 10)	WY OVX (ovariectomized, given Wyeth 14643) (N = 9)	FENO OVX (ovariectomized, given fenofibrate) (N = 10)	PIO OVX (ovariectomized, given pioglitazone) (N = 9)
CORTICAL VOLUME (Ct.V) (μm^3^)	31.98 ± 2.449	28.26 ± 1.491^aa^	28.59 ± 1.825 ^aa^	27.71 ± 2.443 ^aaa^	23.36 ± 1.578 ^aaa, bbb, ccc, ddd^
CORTICAL THICKNESS (Ct.Th) (μm)	449.4 ± 18.80	422.3 ± 21.21^a^	419.5 ± 19.64 ^aa^	426.5 ± 18.48 ^a^	372.7 ± 15.69 ^aaa, bbb, ccc, ddd^
TRABECULAR BONE VOLUME (BV) (μm^3^)	10.94 ± 1.365	10.69 ± 1.291	11.03 ± 1.176	10.25 ± 1.284	10.17 ± 1.468
TOTAL VOLUME (TV) (μm^3^)	20.12 ± 2.062	23.70 ± 2.926 ^a^	23.70 ± 2.926 ^aa^	21.62 ± 1.737	26.52 ± 1.924 ^aaa, b, ccc, d^
TRABECULAR THICKNESS (Tb.Th) (μm)	155.2 ± 6.893	147.2 ± 3.445 ^a ^	146.2 ± 4.298 ^aa^	150.6 ± 4.886	138.9 ± 6.828 ^aaa, b, ccc, d^
CONNECTIVITY DENSITY (CD) (1/mm^3^)	44.42 ± 9.160	46.34 ± 7.634	60.49 ± 20.60	47.26 ± 5.699	46.09 ± 5.317
STRUCTURE MODEL INDEX (SMI) (0.0-3.0)	0.6509 ± 0.2211	0.9811 ± 0.1391 ^a^	0.8556 ± 0.1588	0.9610 ± 0.1824 ^aa^	1.179 ± 0.4074 ^aaa,d^
TRABECULAR BONE VOLUME FRACTION (BV/TV)	0.5436 ± 0.04273	0.4511 ± 0.02421 ^aaa^	0.4700 ± 0.07018	0.4720 ± 0.03425 ^aa^	0.3833 ± 0.05050 ^aaa, b, cc, d^

^a, aa, aaa ^= *p *< 0.05, *p *< 0.01, *p *< 0.001 when compared to SHAM group

^b, bb, bbb ^= *p *< 0.05, *p *< 0.01, *p *< 0.001 when compared to OVX group

^c, cc, ccc ^= *p *< 0.05, *p *< 0.01, *p *< 0.001 when compared to FENO OVX group

^d, dd, ddd ^= *p *< 0.05, *p *< 0.01, *p *< 0.001 when compared to WY OVX group

**Table 2:** Mean malues ± SD of bone architecture parameters in the femoral metaphysis, determined by means of μCT scanning analyses in rats after 4 months of treatment

FEMORAL METAPHYSIS	SHAM (sham-operated controls) (N = 10)	OVX (ovariectomized controls) (N = 10)	WY OVX (ovariectomized, given Wyeth 14643) (N = 9)	FENO OVX (ovariectomized, given fenofibrate) (N = 10)	PIO OVX (ovariectomized, given pioglitazone) (N = 9)
CORTICAL VOLUME (Ct.V) (μm^3^)	28.77 ± 1.773	27.13 ± 1.551 ^a^	28.14 ± 1.222	28.71 ± 1.795	23.37 ± 1.225 ^aaa, bbb, ccc, ddd^
CORTICAL THICKNESS (Ct.Th) (μm)	735.5 ± 22.90	694.4 ± 41.84 ^a^	700.6 ± 21.83 ^aa^	709.9 ± 28.52 ^a^	594.7 ± 39.58 ^aaa, bb, cc, ddd^
TRABECULAR BONE VOLUME (BV) (μm^3^)	6.554 ± 0.8317	5.147 ± 0.5373 ^aa^	5.697 ± 0.6074 ^a^	5.615 ± 1.410	4.996 ± 1.048 ^aa^
TOTAL VOLUME (TV) (μm^3^)	21.85 ± 1.640	25.23 ± 1.734 ^a^	23.65 ± 1.828	23.76 ± 2.448	28.58 ± 2.513 ^aaa, b, ccc, ddd^
TRABECULAR THICKNESS (Tb.Th) (μm)	148.3 ± 5.638	146.7 ± 6.034	144.1 ± 6.402 ^c^	154.8 ± 11.59	141.5 ± 6.146 ^aa, cc^
CONNECTIVITY DENSITY (CD) (1/mm^3^)	14.20 ± 5.740	10.91 ± 2.118	17.91 ± 5.004 ^bb,c^	12.01 ± 4.295	11.56 ± 2.533 ^dd^
STRUCTURE MODEL INDEX (SMI) (0.0-3.0)	0.6682 ± 0.2296	1.596 ± 0.2397 ^aaa^	1.394 ± 0.1283 ^aaa, b^	1.518 ± 0.2138 ^aaa^	1.742 ± 0.2773 ^aaa, dd^
TRABECULAR BONE VOLUME FRACTION (BV/TV)	0.2991 ± 0.03208	0.2056 ± 0.02008 ^aaa^	0.2389 ± 0.02892 ^aaa, b^	0.2340 ± 0.04048 ^aaa^	0.1744 ± 0.02128 ^aaa, bb, cc, ddd^

^a, aa, aaa ^= *p *< 0.05, *p *< 0.01. *p *< 0.001 when compared to SHAM group

^b, bb, bbb ^= *p *< 0.05, *p *< 0.01, *p *< 0.001 when compared to OVX group

^c, cc, ccc ^= *p *< 0.05, *p *< 0.01, *p *< 0.001 when compared to FENO OVX group

^d, dd, ddd ^= *p *< 0.05, *p *< 0.01, *p *< 0.001 when compared to WY OVX group

The FENO OVX group maintained TV and Tb.Th in the femoral head and the Ct.V, BV and TV in the metaphysis at SHAM levels, in contrast to the OVX control group (Table 1 and 2). However, Ct.Th and BV/TV ratio in both the femoral head and metaphysis were significantly lower in the FENO OVX group compared to the SHAM group (Tables 1 and 2).

The PIO OVX group had significantly lower Ct.V, Ct.Th, and BV/TV in both the femoral head and the metaphysis, and also the highest EV, compared to all other groups (Tables 1 and 2). In addition, the PIO OVX group had significantly lower BV in the metaphysis than the SHAM group, and significantly reduced Tb.Th in the femoral head compared to the other groups (Tables 1 and 2).

There was no difference in CD between the groups, except in the WY OVX group, which showed significantly higher CD in the femoral metaphysis, compared to the FENO OVX and OVX groups (Table 2). All the ovariectomized groups exhibited significantly higher SMI than the SHAM group, both in the femoral head and metaphysis, except for WY OVX in the femoral head (Tables 1 and 2).

In conclusion, ovariectomized rats given fenofibrate, and to some extent rats given Wyeth 14643, partly maintained bone architectural structures at sham levels in the femur, while ovariectomized rats given pioglitazone had further deterioration of femoral bone architecture.

### Biomechanical testing

The FENO OVX and WY OVX groups maintained the ultimate bending moment and energy absorption at sham levels in the femoral neck (Table 3). The OVX controls had significantly lower bending moment and energy absorption in the femoral neck compared to SHAM, while the PIO OVX group had significantly lower bending moment and energy absorption than all the other groups except OVX controls in the femoral neck, and all the other groups for the femoral neck (Table 3). There was no significant difference between the groups regarding stiffness, neither in the femoral neck nor shaft (Table 3).

**Table 3:** Mechanical properties of the femoral neck and shaft in mean values ± SD after 4 months of treatment

	SHAM (sham-operated controls) (N = 10)	OVX (ovariectomized controls) (N = 10)	WY OVX (ovariectomized, given Wyeth 14643) (N = 9)	FENO OVX (ovariectomized, given fenofibrate) (N = 10)	PIO OVX (ovariectomized, given pioglitazone) (N = 9)
ULTIMATE BENDING MOMENT (Nm) Femoral neck Femoral shaft	63.0 ± 8.9 81.7 ± 6.3	52.1 ± 8.9 ^a^ 76.8 ± 7.6	62.0 ± 12 79.4 ± 3.7	56.5 ± 9.8 75.0 ± 4.9	47.6 ± 8.2 ^a,c,dd^ 65.4 ± 7.2 ^aaa,bb,c,ddd^
ENERGY ABSORPTION (J × 10^-2^) Femoral neck Femoral shaft	13.5 ± 2.7 15.3 ± 2.3	9.94 ± 2.6 ^a^ 13.3 ± 2.8	13.8 ± 3.7 ^b^ 13.9 ± 1.4	12.0 ± 3.7 14.3 ± 2.0	8.60 ± 2.2 ^aaa,c,dd^ 10.0 ± 3.2 ^aa,b, cc,d^
ULTIMATE BENDING STIFFNESS (Nm/° × 10^-2^) Femoral neck Femoral shaft	370 ± 58 574 ± 61	317 ± 53 582 ± 58	359 ± 81 586 ± 66	344 ± 49 550 ± 35	339 ± 83 540 ± 81

^a, aa, aaa ^= *p *< 0.05, *p *< 0.01, *p *< 0.001 when compared to SHAM group

^b, bb, bbb ^= *p *< 0.05, *p *< 0.01, *p *< 0.001 when compared to OVX group

^c, cc, ccc ^= *p *< 0.05, *p *< 0.01, *p *< 0.001 when compared to FENO OVX group

^d, dd, ddd ^= *p *< 0.05, *p *< 0.01, *p *< 0.001 when compared to WY OVX group

### Plasma osteocalcin, CTx, leptin and adiponectin

**Figure 3:**
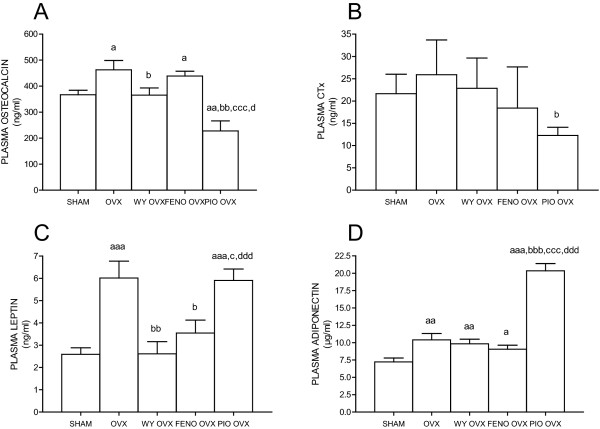
**Osteocalcin (**A**), CTx (**B**), leptin (**C**) and adiponectin (**D**) in plasma**. Rats were either sham operated and given vehicle (SHAM) or ovariectomized and given vehicle (OVX), Wyeth 14643 (WY OVX), fenofibrate (FENO OVX) or pioglitazone (PIO OVX) respectively. Data are presented as means ± SD. ^a,aa,aaa ^= *p *< 0.05, *p *< 0.01, *p *< 0.001 compared SHAM. ^b,bb, bbb ^= *p *< 0.05, *p *< 0.01, *p *< 0.001 compared to OVX. ^c,ccc ^= *p *< 0.05, *p *< 0.001 compared to FENO OVX group. ^d,ddd ^= *p *< 0.05, *p *< 0.001 compared to WY OVX.

Plasma osteocalcin levels were significantly higher in the OVX (463 ± 36 ng/ml, *p *= 0.0291) and FENO OVX groups (438 ±19 ng/ml, *p *= 0.0119) compared to the SHAM group (367 ± 17.5 ng/ml) (Figure 3A). The WY OVX group (365 ± 84 ng/ml) had significantly lower osteocalcin levels than the OVX group (*p *= 0.05), but did not differ from the SHAM group (Figure 3A). The PIO OVX rats had significantly lower plasma osteocalcin than all other groups (256 ± 29 ng/ml, *p *= 0.0033 vs. SHAM, *p *= 0.0114 vs. OVX, *p *< 0.0001 vs. FENO OVX and *p *< 0.0001 vs. WY OVX) (Figure 3A).

Plasma CTx levels were significantly lower in the PIO OVX group (12.3 ± 1.8 ng/ml, *p *= 0.0006) compared to the OVX group (21.7 ± 4.4 ng/ml) (Figure 3B). There were no significant differences in CTx levels between any of the other groups (SHAM 25.9 ± 2.5 ng/ml, FENO OVX; 18.4 ± 2.9 ng/ml, WY OVX; 22.8 ± 6.8 ng/ml) (Figure 3B).

Plasma leptin levels were significantly higher in the OVX (6.0 ± 0.3 ng/ml, *p *= 0.0006) and PIO OVX groups (5.9 ± 1.4 ng/ml, *p *= 0.0008) compared to the SHAM group (2.6 ± 0.3 ng/ml) (Figure 3C). There was no difference in the plasma leptin levels between the FENO OVX (3.5 ± 1.7 ng/ml), WY OVX(2.6 ± 1.7 ng/ml) and SHAM groups, but the FENO OVX and WY OVX groups had significantly lower plasma leptin than the OVX group (*p *= 0.0106 and *p *= 0.0022, respectively) (Figure 3C).

Plasma adiponectin levels were significantly increased in all ovariectomized groups (OVX; 10.4 ± 0.6 μg/ml, *p *= 0.0081, FENO OVX; 9.1 ± 0.6 μg/ml, *p *= 0.0338, WY OVX; 9.8 ± 2.0 μg/ml, *p *= 0.0077, PIO OVX; 20.4 ± 1.1 μg/ml, *p <*0.0001) compared to the SHAM group (7.2 ± 0.6 μg/ml) (Figure 3D). The PIO OVX group had also significantly higher plasma adiponectin levels compared to the OVX, FENO OVX and WY OVX groups (*p *< 0.0001 for all) (Figure 3D).

### Effect of fenofibrate and pioglitazone on differentiation of rat bone marrow cells

**Figure 4:**
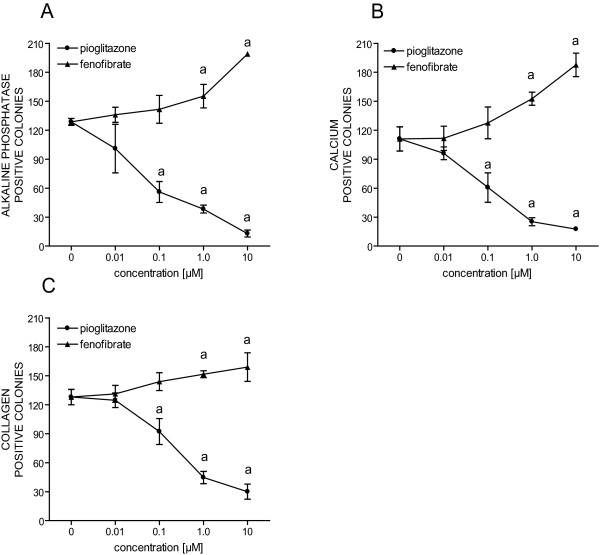
**Effects of fenofibrate and pioglitazone on osteoblast differentiation**. Rat primary bone marrow cells were treated with increasing doses of PPAR agonists for 21 days and stained sequentially for ALP (**A**), calcium (**B**) and collagen (**C**). ^a ^= *p *< 0.05 compared with vehicle treated cells.

The PPARα agonist fenofibrate (1.0 and 10 μM) significantly increased the number of ALP (+20-55%), calcium (+37-70%) and collagen (+18-26%)-positive colonies in rat bone marrow cells examined with the mCFU-f assay (Figure 4). On the other hand, the PPARγ agonist pioglitazone (0.1, 1.0 and 10 μM) significantly reduced the number of ALP (-57-90%), calcium (-45-85%) and collagen (-30-77%)-positive colonies (Figure 4). Also, pioglitazone led to adipocyte forming colonies, with clearly visual formation of lipid droplets (data not shown).

## Discussion

Our study provides the first evidence that administration of the PPARα agonists fenofibrate and Wyeth 14643 to a certain level can maintain bone quality at sham levels in an ovariectomized rat model. Furthermore, this study differentiates the PPARα agonists from the PPARγ agonists regarding skeletal effects, since the PPARγ agonist pioglitazone exerts the opposite effect by further boosting ovariectomy-induced bone loss in rats.

Ovariectomized control rats had significantly lower gain in femoral BMC and BMD associated with reduced biomechanical strength parameters compared to sham-operated controls. The relatively young age of the rats may explain why the differences found between the SHAM and OVX controls were less pronounced than expected. According to Kahrode et al, 2008 [46], rats from 2-15 months are applied in the rat OVX model, and while use of older animals is attractive due to steady bone turnover rate, the use of young adult rats can also provide consistent, reproducible and interpretable results. Ovariectomy of skeletally immature rats results in achievement of a lower peak bone mass (total bone mass present at the end of the skeletal maturation, which for rats are considered to occur between 47-61 weeks of age [47]).

In our study, fenofibrate had a partly preventive effect on bone in this rat model of osteoporosis, as femoral and whole body BMC and BMD were maintained at the same levels as for sham-operated rats, and this group also exhibited significantly higher femoral BMC and BMD than ovariectomized controls. Fenofibrate also prevented deterioration of the bone architecture to a certain level, as reflected in maintenance of trabecular BV, trabecular thickness Tb.Th, and TV. We have previously shown that fenofibrate increased femoral BMD and reduced bone medullary area in intact female rats [12,13] suggesting an inhibition of endosteal bone resorption. Chan *et al. *found that fibrates directly inhibit human osteoclast activity and function [22], and Okamoto *et al. *demonstrated that fenofibrate suppresses osteoclast differentiation by inhibiting NFκB-signaling [48]. We have, however, not been able to confirm a direct effect of fenofibrate on human osteoclasts [13]. This is consistent with the findings of Still *et al*., who showed that administration of bezafibrate to rats caused no change in either the number of osteoclasts or the serum levels of resorption markers [9].

In contrast to the ovariectomized control group, rats given fenofibrate and Wyeth 14643 had similar bending moment and energy absorption as the sham group at the femoral neck, indicating maintained mechanical strength in spite of the ovariectomy.

Furthermore, administration of fenofibrate and Wyeth 14643 resulted in maintenance of femoral BMC at the same level as the sham control group.

We have previously shown that fenofibrate stimulates osteoprotegerin (OPG) release from the mouse preosteoblast cell line MC3T3-E1 [13], and it has also been found to enhance plasma OPG in humans [49]. These findings suggest an antiresorptive effect of fenofibrate.

In a previous study, we demonstrated that fenofibrate elevated plasma osteocalcin levels in intact female rats [13]. In the present study, however, the plasma osteocalcin levels were similar in the ovariectomized group and the fenofibrate group, and both groups had elevated osteocalcin levels compared to the sham-operated group. It is therefore difficult to differentiate if the elevated level of plasma osteocalcin level in the fenofibrate group is due to increased bone formation or increased bone turnover associated with ovariectomy.

We and others have previously shown that activation of PPARα increases osteoblast gene expression of differentiation markers [13], increases ALP activity, induces osteoblastic maturation and matrix calcification [50] in MC3T3-E1 cells. In the present study we found that fenofibrate significantly enhanced the number of ALP-, calcium- and collagen-positive colonies in bone marrow cells from rats.

Still *et al. *showed that bezafibrate and linoleic acid increased BMD and periosteal bone formation in male rats [9]. Since the most pronounced effect was found for linoleic acid, which preferentially is a PPARδ agonist, they concluded that the effects were mediated through PPARδ activation. Wyeth 14643 and fenofibrate possess high and equal affinity to PPARα [51], and we demonstrate that administration of these PPARα agonists to some level maintain bone mass and biomechanical at sham levels.

The PIO OVX group exhibited enhanced bone loss and impaired bone architecture, reflected in a significant decrease in cortical volume and thickness in both femoral head and shaft, and an elevated total volume. In accordance with these findings, we also found reduced mechanical strength in this group. These results are in agreement with several earlier studies, all showing reduced bone formation and/or a decrease in bone mass, in rodents treated with glitazones, mainly rosiglitazone [24-29]. Similar findings were recently demonstrated in humans [30-33,52,53]. Pioglitazone is still used for treatment of diabetes mellitus 2, while rosiglitazone has been withdrawn due to adverse effects [54]. We show that pioglitazone causes negative skeletal effects similar to those seen for rosiglitazone. Plasma levels of osteocalcin were significantly reduced in the PIO OVX group compared to all the other groups, suggesting that the negative effect of PPARγ agonists on the skeleton is caused by inhibition of bone formation, rather than elevated bone resorption. This is supported by the fact that plasma CTx was significantly lower in the rats receiving pioglitazone. Pioglitazone also reduced the number of ALP-, calcium- and collagen -positive colonies *in vitro *in bone marrow cells from rats. This is in accordance with several other studies, showing that pioglitazone [55] and the PPARγ agonist BRL-49653 [56] stimulate adipogenesis, inhibit osteogenesis and reduce ALP activity in mesenchymal cells from mouse and rat bone marrow, respectively. We have previously studied the effect of pioglitazone on human osteoclast differentiation and activity [13], but could not detect any effect with the doses used in our study (0.1-10 μM). Studies regarding the effects of PPARγ agonists on osteoclasts are contradictory; some studies report an inhibition of osteoclast differentiation and activity [20,21,57], while others demonstrate a pro-osteoclastogenic effect of PPARγ [22,58]. Since we could not demonstrate a direct effect of either fenofibrate or pioglitazone on osteoclasts [13], we speculate whether the observed skeletal effects of PPAR agonists mainly are mediated through the regulation of mesenchymal cells within the bone marrow.

The doses of rosiglitazone used in previous rodent studies were 3.0-25 mg/kg/day, whereas we used a higher daily dose of pioglitazone (35 mg/kg). Rosiglitazone is a more potent PPARγ agonist than pioglitazone [4], and several studies have estimated comparable glycemic effects of rosiglitazone 2-8 mg with pioglitazone 15-45 mg, respectively [59-61].

Most studies of glitazones treatment in rodents [24-29], also report enhanced bone marrow adiposity, supporting evidence from *in vitro *studies in which PPARγ activation in bone cells was found to promote adipogenesis at the expense of osteoblastogenesis [14-16,18,62]. We did not measure the fat content in bone marrow; the PIO OVX group, however, displayed significantly higher body fat mass and lower % lean mass than all the other groups, showing that PPARγ activation stimulates adipogenesis in general.

The FENO OVX group maintained % lean mass at sham levels in contrast to the ovariectomized controls, which is in accordance with our previous study where fenofibrate increased lean mass in intact female rats [13].

PPARα activation is found to induce hepatomegaly in rodents [8] and in accordance with this we found increased liver weights in the FENO and WY groups. This was not examined any further, as this was beyond the scope of this study.

The skeletal effects of PPAR agonists may also be indirect, for instance through regulation of adipokine production in adipose tissue. There is a connection between the amount of body fat and bone mass as recently reviewed by several authors [63-65]. Adipose tissue produces several hormones assumed to be involved in the regulation of bone metabolism, such as adiponectin [66,67] and leptin [68,69]. However, there are conflicting results concerning the skeletal effects of adipokines [63-65].

In our study we found significant differences in plasma leptin and adiponectin levels between the groups, especially the PIO OVX group exhibited higher levels than the others. In humans, circulating leptin concentrations correlate with body mass index (BMI) and the total amount of body fat [70,71]. We found that the WY OVX and the FENO OVX groups had similar plasma leptin levels as SHAM, despite a significantly higher body weight and fat mass. Adiponectin is known to stimulate insulin sensitivity, and usually correlates negatively with increased fat mass [72-74]. In spite of the large enhancement in fat mass, the PIO OVX group exhibited the highest level of plasma adiponectin among the groups. This is in accordance with previous studies demonstrating that glitazones increase circulating adiponectin levels in humans as well as rodents [75-77]. It is difficult to elaborate whether these differences explain the various skeletal effects of PPARα and PPARγ agonists.

## Conclusion

We present evidence for a positive effect of PPARα agonists on bone. Fenofibrate and Wyeth 14643 maintained to a certain level bone mass, some parameters of femoral bone architecture and mechanical strength in the femoral shaft at sham levels, in spite of ovariectomy. We also confirm results from previous studies showing major negative skeletal effects of PPARγ agonists.

Fenofibrate is currently used to treat hyperlipidemia, and our results indicate that fenofibrate could be beneficial for the skeleton for patients on treatment.

## Competing interests

The authors declare that they have no competing interests.

## Authors' contributions

AKS helped in design, conduct/data collection, analysis, statistics and writing of the manuscript. IW, BIG, RF, JHW, and CP helped in conduct/data collection, EFE, JER and US helped in design and writing. All authors have read and approved the final manuscript.

## Pre-publication history

The pre-publication history for this paper can be accessed here:

http://www.biomedcentral.com/1472-6823/11/11/prepub
